# Postpartum vitamin A supplementation for HIV-positive women is not associated with mortality and morbidity of their breastfed infants: evidence from multiple national surveys in sub-Saharan Africa

**DOI:** 10.1186/s12887-020-02131-8

**Published:** 2020-05-13

**Authors:** Samson Gebremedhin

**Affiliations:** School of Public Health, Addis Ababa University, Addis Ababa, Ethiopia

**Keywords:** Vitamin a supplementation, HIV, Infant mortality, Fever, Diarrhoea, Cough, Lactation, Demographic and health surveys, Sub-Saharan Africa

## Abstract

**Background:**

Vitamin A supplementation (VAS) in the postpartum period improves the vitamin A concentration of breast milk and vitamin A status is an important predictor of childhood survival. It is also known that Vitamin A Deficiency (VAD) is more prevalent in HIV-infected women. This study investigated the association between vitamin A supplements provided to HIV-positive women in the postpartum period and mortality and morbidity of their breastfed infants in sub-Saharan Africa (SSA) where the prevalence of VAD and HIV is high.

**Methods:**

This cross-sectional study was conducted based on the secondary data of 838 HIV-positive women (309 vitamin A supplement and 529 non-supplemented) extracted from the datasets of 43 Demographic and Health Surveys (DHS) conducted in 26 SSA countries between 2003 and 2015. The data of HIV-positive women who gave a live birth in the preceding 6 months of the survey and who were breastfeeding their infants at the time of the survey or who breastfed their deceased infants until the time of death, were included in the analysis. The association of postpartum VAS with early infant mortality (death in the first 6 months of birth) and morbidity secondary to fever, diarrhoea and cough with respiratory difficulties in the preceding 2 weeks was assessed by mixed-effects logistic regression model and interpreted using adjusted odds ratio (AOR) with the 95% confidence intervals (CI).

**Results:**

About one-third (36.9%) of the HIV-positive women received VAS soon after the recent delivery. The early infant mortality rate per 1000 live births in vitamin A supplemented group was 100 (95% CI: 67–133) and the corresponding level for non-supplemented group was 125 (95% CI: 97–154). Yet, in the multivariable model adjusted for seven potential confounders, the association was not significant (AOR = 1.10: 95% CI, 0.57–2.13). Similarly, postpartum VAS was not significantly associated with the occurrence of cough with difficult breathing (AOR = 0.65: 95% CI, 0.39–1.10), diarrhoea (AOR = 0.89: 95% CI, 0.50–1.58) and fever (AOR = 1.19: 95% CI 0.78–1.82) in their breastfed infants.

**Conclusion:**

VAS provided to HIV-positive women in the immediate postpartum period does not have significant association with the mortality and morbidity of their breastfed infants.

## Background

Vitamin A deficiency (VAD) is a major public health problem in many low- and middle-income countries. Globally more than 120 countries have moderate or severe public health significance of VAD as measured by biochemical insufficiency in pre-school children [1]. Globally, low serum retinol concentration affects 33% of children and 15% of pregnant women. Especially South-East Asia and sub-Saharan Africa (SSA) regions have the highest burden of VAD [1]. Established consequences of VAD among young children include increased risk of mortality and severity of infections, blindness, growth retardation and anemia [1]. Similarly, during pregnancy VAD predisposes to anemia, clinical infections and night blindness [2].

Vitamin A supplementation (VAS) is a proven, quick and low-cost strategy for correcting vitamin A status of populations [3]. Systematic reviews of randomized controlled trials suggested beyond doubt that in children 6–59 months of age, VAS reduces all-cause mortality by 25% and significantly diminishes occurrence of diarrhoea, measles and xeropthalmia [4, 5]. Furthermore, neonatal VAS may marginally reduce 6-month infant mortality in setting where the magnitude of VAD is high [6]. In many low-income countries routine VAS is already in place for combating the deficiency in preschool children and lactating women. Children 6–59 months receive biannual and high dose (100,000–200,000 International Unit (IU)) supplements and lactating women are provided with a single 200,000 IU supplement within 6 weeks postpartum.

In women, breastfeeding increases the requirement for vitamin A and the amount lost through lactation may predispose to maternal VAD [7]. In the first 6 months of life, breastfed infants consume more than 300 μmol of vitamin A from the mother’s milk [8]. Breast milk vitamin A concentration is sensitive to maternal dietary intake and in the situation of inadequate intake, the infant may not get enough in the breast milk [9]. Convincing evidence exists that single high dose VAS (60–120 mg retinol equivalent) after giving births improves the retinol concertation of breast milk at 3–3.5 months postpartum [9]. Yet, systematic reviews have suggested that supplement provided in the first 6 weeks of birth does not significantly reduce maternal and infant mortality and morbidity [9, 10].

HIV/AIDS remains a major global public health threat. In 2018 about 38 million people were living with HIV and the SSA is the most seriously affected region accounting for approximately 70% of the existing cases [11, 12]. Complex relationship exists between malnutrition and HIV infection. HIV compromises nutrition through multiple pathways including reducing appetite, causing malabsorption of nutrients, altering metabolism and increasing the demand for essential nutrients. Further, HIV-related immune impairment may predispose to secondary malnutrition. Advanced HIV infection causes wasting syndrome and compromises economic productivity and food security [13, 14]. HIV infection increases energy requirements by 10 to 30% depending on the stage of progression of the infection [15].

It has been reported that VAD is more common in HIV-infected women than in uninfected women [16–18]. Further, a couple of studies witnessed increased mortality of infants born to vitamin A-deficient HIV-positive mothers [19, 20]. Consequently, this study explored whether receipt of VAS by HIV-positive women in the postpartum period is associated with reduction in mortality and morbidity of their breastfed infants or not. The study was conducted based on the secondary data of multiple Demographic and Health Surveys (DHS) carried out in SSA region where the magnitudes of VAD, HIV-infection and infant mortality are all high. In general, at the beginning of the study it was hypothesized that postpartum VAS to HIV-positive women would be associated with reduced mortality and morbidity of their breastfed infants based on the following propositions (i) HIV-positive women and their new-borns are at increased risk of VAD [16–18]; (ii) postpartum VAS improves vitamin A concentration of breast milk [9]; and (iii) vitamin A reduces the risk of child mortality and morbidity in settings where VAD is prevalent [4, 5].

## Methods

### Study design

This cross-sectional observational study was conducted based on the secondary data of 43 DHS carried out in 26 SSA countries between 2003 and 2015. Demographic and Health Surveys are nationally-representative household cross-sectional surveys being implemented on regular basis in many low- and middle-income countries by national agencies with the support of the Measure-DHS Program. The surveys are intended to provide updated information on a wide range of population and health indicators. In many countries the DHS are typically implemented in 5 years interval.

Pertaining to the inclusion and exclusion criteria, the geographical scope of the study was delimited to the SSA region considering the fact that VAD, HIV-infection and infant mortality are all highly prevalent in the sub-continent. In the surveys conducted before 2003 and after 2015, HIV status and postpartum VAS-related data respectively, had not been collected in women of reproductive age (15–49 years); thus, the surveys were excluded from the study. The list of the surveys considered eligible for the analysis is provided as a supplementary file (Supplementary file 1).

**Fig. 1:**
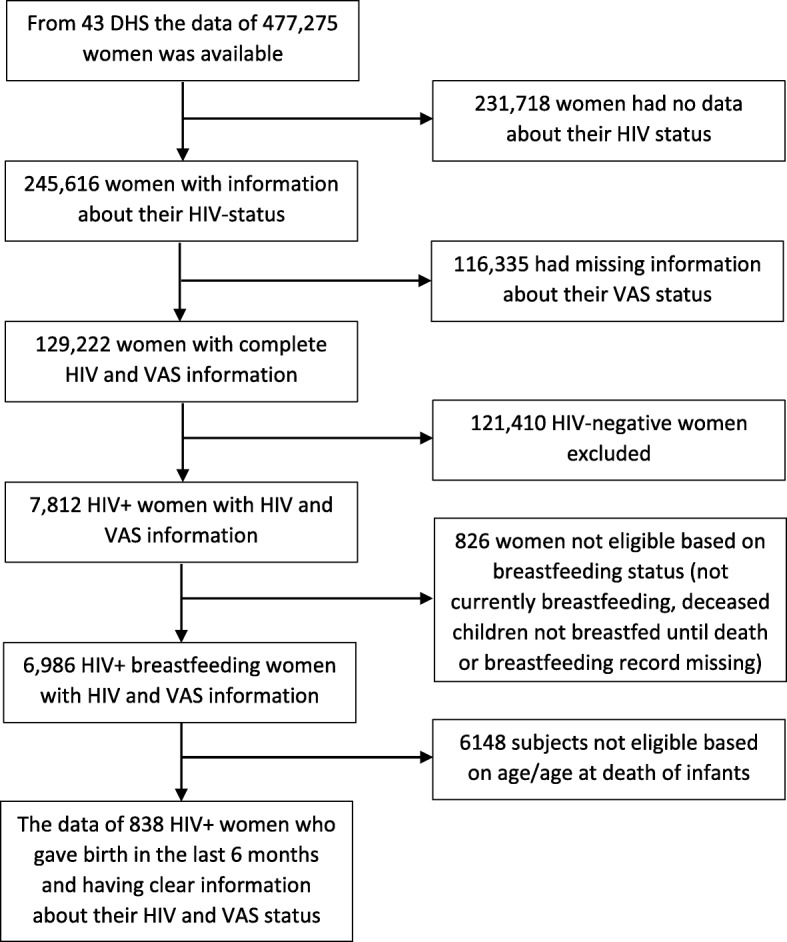
Flow chart of the study

The datasets of the 43 surveys were accessed from the Measure DHS website (https://dhsprogram.com/data/) and the information about non-eligible subjects (HIV-negative women, women who have no information about HIV and VAS status and those who did not give birth within 6 months of the survey) was dropped. Ultimately, the data of 838 HIV-positive women who gave live birth in the preceding 6 months, who have clear information about their postpartum VAS exposure status and who were breastfeeding their infants at the time of the survey (or until the death of their deceased infants) retained in the analysis (Fig. 1).

For each eligible subject relevant information including VAS status, survival status of the infant, age at death for deceased infants, occurrence of diarrhoea, fever and cough with shortness of/difficult breath (proxy for acute respiratory infection (ARI)) in the preceding 15 days of the survey, basic socio-demographic characteristics and other potential confounders including access to mass-media, health service utilization, types of drinking water source and household sanitary facility, maternal anthropometry were extracted.

### Sample size and power

As the study was conducted based secondary data, sample size determination has not been made. Yet, post-hoc power calculation indicated, the available sample size of 838 HIV-positive women – comprising 309 vitamin A supplement and 529 non-supplemented subjects – is sufficient to detect 6% difference in early infant mortality (death in the first 6 months of birth) between the two group with approximately 80% power and 95% confidence level. The post-hoc power calculation was made assuming that the early Infant Mortality Rate (IMR) in non-supplemented group is 125 per 1000 live births.

### Sampling approach of DHS

Demographic and Health Surveys are designed to generate representative data at national and sub-national (region or state) levels and typically use a two-stage cluster sampling approach for recruiting the study participants. At the first stage, a sample of Enumeration Areas (EAs) stratified by sub-national regions and place of residence (urban, rural) is selected with probability proportional to size approach. In the selected EAs an exhaustive listing of households is performed. At the second stage, a predetermined 20 to 30 households is selected by systematic sampling approach. In each selected household, all eligible subjects including women of reproductive age are identified, interviewed and blood samples for HIV testing are collected [21].

### Data collection procedures of DHS

In the original surveys, data were collected from the respondents by trained interviewers using standardized and pretested questionnaires prepared in the major local languages of the respective host countries. Maternal receipt vitamin A supplement in the post-partum period was assessed by showing a vitamin A capsule to the study participant and asking whether she had taken the same soon after the recent delivery or not. The occurrence of diarrhoea, fever and cough with shortness or difficult breath was assessed by asking one-by-one if the index infant had the same problem in the preceding 2 weeks of the survey without any further clinical evaluation. Breastfeeding practice was assessed by asking whether the mother breastfed her child in the preceding day of the survey or not, irrespective of frequency or amount of breastfeeding.

Maternal height and weight were measured using calibrated tools, body-mass-index (BMI) was computed using the standard formula and women were classified as thin (BMI < 18.5 kg/m^2^), normal (BMI between 18.5 and 24.9 kg/m^2^) or overweight/obese (BMI > 25 kg/m^2^). Birthweight of the infants were determined based on recall of the mothers and classified as low (< 2.5 kg), normal (2.5–3.9 kg) or macrosomic (4.0 or above kg) birthweight.

### Data management and analysis

The datasets of the 43 surveys were downloaded from the Measure DHS website in SPSS format and merged into one spreadsheet. Irrelevant variables and data of non-eligible subjects were dropped and the remaining data got cleaned and recoded as needed. The dataset analysed is provided as a supporting file (Supplementary file 2).

Data were analysed using weight analysis approach on the basis of the sample weights readily available in the datasets. Data were presented using appropriate measures of central tendency and dispersion, frequency distributions and tables. Wealth index, a composite index of living standard, was determined based on ownership of valuable household assets (such as television, radio and mobile phone), materials used for housing construction (type of floor, wall and roof) and types of water source and sanitation facility. The analysis was made using Principal Component Analysis, ultimately a factor with the highest explained variability based on eigenvalue of 1 was identified and was categorized into wealth quintiles (poorest, poorer, middle, richer, richest). Wealth index was determined separately for each survey and pooled into one from all surveys.

The association of postpartum VAS with early infant mortality and infant morbidity secondary to fever, diarrhoea and ARI-related symptoms in the preceding 2 weeks was assessed using mixed-effects bivariable and multivariable logistic regression models with random slope for each country. Separate models were developed for each of the aforementioned four outcome variables. The vitamin A supplemented and non-supplemented groups were initially compared based on multiple socio-demographic, health service utilization and access to mass media-related variables using Pearson’s Chi-square test. Variable that were found to be significantly unbalanced (*p*-value < 0.05) or marginally unbalanced (*p*-value between 0.2 and 0.05) were considered as potential confounders; thus, got adjusted in the multivariable models. The fitness of the multivariable models was assessed using Hosmer and Lemeshow test. Interpretation was made by exponentiating the logistic regression coefficients into crude (COR) and adjusted (AOR) odds ratios.

### Ethical consideration

The datasets were downloaded after securing permission from the Measure DHS Program. For this specific secondary data analysis ethical clearance was not sought. Nevertheless, all the original DHS protocols were reviewed and approved by the Demographic and Health Survey Program, ICF International Inc., Institutional Review Board.

## Results

### Basic characteristics of the respondents

The data of 838 HIV-positive women who gave live birth in the preceding 6 months were included in the analysis. About one-third (309 (36.9%)) of the mothers received vitamin A supplement after the recent birth; whereas, the remaining two-thirds (529 (63.1%)) did not. Most of the study subjects (70.2%) were from the southern Africa region and smaller proportions (< 10%) were drawn from the eastern or central parts of Africa.

Nearly two-thirds (64.8%) of the respondents were selected from male-headed households and 40.5% were from households of richer or richest wealth quintiles. The mean (± standard deviation) age of the respondents was 27.9 (±6.0) years and about half (52.3%) were between 25 to 34 years of age. About two-fifths (41.4%) had secondary or post-secondary education and 64.0% resided in rural areas. Three-quarters (73.4%) were married or living together with their partners. Nearly two-thirds of the women had normal BMI (18.5–24.9 kg/m^2^).

Table 1 compares the geographic distribution, basic socio-demographic characteristics, anthropometric characteristics, patterns of health service utilization and access to mass media between vitamin A supplemented and non-supplemented groups using chi-square test. In terms of socio-demographic characteristics, the two groups were balanced (*p* > 0.05) in most of the characteristics including source of drinking water and household sanitation facility. However, infants born to vitamin A supplemented women were significantly older than their counterparts (2.9 ± 1.6 mos vs 2.6 ± 1.7 mos) (*P = 0.017*). Regarding, utilization of preventive health services, women who were vitamin A supplemented had better utilization of health facility delivery, postnatal care and childhood vaccination services (*P < 0.001*). Significant different in the patten of birthweight was also observed between the two groups (*p* = 0.001). No meaningful differences were observed in terms of access to mass media including frequency of watching television and listening to radio (Table 1).

**Table 1:** Basic characteristics of HIV-positive women included in the analysis, Sub-Saharan Africa, 2003–2015

Variables (***n*** = 838)	Vitamin A supplemented				Both (***n*** = 838)		***P***-value
	Yes (***n*** = 309)		No (***n*** = 529)				
	Freq	%	Freq	%	Freq	%	
Sub-Saharan Africa Region							
Eastern	10	3.4	52	9.9	63	7.5	< 0.001*
Southern	232	74.8	357	67.4	588	70.2	
Western	57	18.5	86	16.2	143	17.0	
Central	10	3.3	35	6.5	45	5.3	
Sex of the household head							
Male	193	62.5	350	66.1	543	64.8	0.279
Female	116	37.5	179	33.9	295	35.2	
Household wealth index							
Poorest or poorer	123	39.6	206	38.9	328	39.1	0.755
Middle	59	19.1	112	21.2	171	20.4	
Richer or richest	128	41.3	211	40.0	339	40.5	
Maternal age (years)							
15–24	105	33.9	162	30.6	267	31.8	0.611
25–34	157	50.7	282	53.2	438	52.3	
35 or above	48	15.4	86	16.2	133	15.9	
Marital status							
Never in union	54	17.4	72	13.6	126	15.0	0.388
Married/living with partner	217	70.2	398	75.2	615	73.4	
Widowed	11	3.5	18	3.4	29	3.5	
Divorced or separated	28	8.9	41	7.8	69	8.2	
Place of residence							
Urban	117	37.9	184	34.9	302	36.0	0.380
Rural	192	62.1	344	65.1	537	64.0	
Maternal education							
No formal education	53	17.2	75	14.2	128	15.3	0.469
Primary education	134	43.4	229	43.4	364	43.4	
Secondary or higher education	122	39.5	224	42.4	347	41.3	
Number of children under the age of 5 years							
0	17	5.4	48	9.0	65	7.7	0.108
1	121	39.0	182	34.4	303	36.1	
2 or more	172	55.5	299	56.6	471	56.2	
Maternal body-mass-index (kg/m^2^)							
< 18.5	22	7.3	31	5.9	53	6.4	0.407
18–5-24.9	200	64.8	369	69.8	569	67.9	
25 or above	80	25.8	122	23.1	202	24.1	
Missing	7	2.2	7	1.3	14	1.6	
Sex of the child							
Boy	158	51.2	239	45.1	397	47.4	0.096
Girl	151	48.8	290	54.9	441	52.6	
Age of the index child (months) (*n* = 737)^×^							
0–1	61	21.6	156	34.1	217	29.4	0.001*
2–3	112	39.6	139	30.6	251	34.0	
4–5	109	38.7	161	35.3	270	36.6	
Birth weight as reported by the mother							
< 2.5 kg	36	11.5	32	6.0	68	8.0	0.001*
2.5–3.9 kg	188	60.9	290	54.8	478	57.1	
4.0 kg or above	12	4.0	26	4.9	38	4.6	
Not weighted at birth or don’t know	73	23.6	181	34.3	254	30.3	
Drinking water source							
Improved	221	71.3	372	70.4	593	70.7	0.766
Unimproved	89	28.7	157	29.6	246	29.3	
Sanitation facility							
Improved	144	27.3	99	31.9	243	29.0	0.146
Unimproved	385	72.7	211	68.1	595	71.0	
Place of delivery							
Home	71	23.1	191	36.1	262	31.3	< 0.001*
Health facility	238	76.9	338	63.9	576	68.7	
Any postnatal check-up by health professional							
No	150	48.4	391	73.8	540	64.4	< 0.001*
Yes	160	51.6	138	26.2	298	35.6	
Child ever vaccinated (*n* = 737)^×^							
No	35	11.4	119	22.4	154	20.9	< 0.001*
Yes	246	79.6	337	63.8	583	79.1	
Frequency of watching TV							
Not at all	188	60.9	354	67.0	543	64.7	0.181
Less than once a week	35	11.3	46	8.6	81	9.6	
At least once a week	86	27.7	129	24.4	215	25.6	
Frequency of listening to radio							
Not at all	110	35.6	165	31.2	275	32.8	0.409
Less than once a week	49	15.8	86	16.3	135	16.1	
At least once a week	150	48.6	278	52.5	428	51.1	
^×^ excluding deceased infants							

### Maternal vitamin a supplementation and survival of breastfed infants

The early infant mortality rate (eIMR) in the entire HIV-positive subjects included in the analysis was 116 (95% CI: 94–137) per 1000 live births. The mortality rate in vitamin A supplemented group was 100 (95% CI: 67–133) and the corresponding rate for non-supplemented group was 125 (95% CI: 97–154) per 1000 live births. However, in the multivariable model that adjusted for seven potential confounders (geographic region of the country, place of delivery, utilization of postnatal care, sex of the newborn, type of household sanitation facility, number of under five children in the household and frequency of watching television) the association was not statistically significant (AOR = 1.10: 95% CI, 0.57–2.13) (Table 2).

**Table 2:** Association between maternal vitamin A supplementation and early infant mortality in HIV-positive women, sub-Saharan Africa

Vitamin A supplementation status	Survival status				Odds ratio	
	Deceased		Alive		Crude	Adjusted^a^
	Freq	%	Freq	%		
Supplemented (*n* = 319)	32	10.0	287	90.0	0.78 (0.50–1.22)	1.10 (0.57–2.13)
Non-supplemented (*n* = 519)	65	12.5	454	87.5	1	1

^a^ Adjusted for geographic region of the country, place of delivery, utilization of postnatal care, sex of the newborn, type of household sanitation facility, number of under five children in the household and Frequency of watching television

### Maternal vitamin a supplementation and morbidity of breastfed infants

Table 3 presents the association between maternal vitamin A supplementation in HIV-positive women and occurrence of diarrhoea, fever and ARI-related symptoms in their breastfed offspring younger than 6 months of age. Among infants of women who received VAS soon after birth, 9.1% of had cough with shortness/difficulty of breath in the preceding 2 weeks of the survey and the corresponding figure was 12.3% in the infants born to non-supplemented women. However, in the multivariable model adjusted for nine potential confounders (geographic region of the country, place of delivery, utilization of postnatal care, sex of the newborn, type of household sanitation facility, number of under five children in the household, frequency of watching television, age of the child and vaccination status of the child), the difference was marginally insignificant (AOR = 0.65: 95% CI, 0.39–1.10) (*p* = 0.108). Likewise, maternal VAS was not associated with reduced odds of diarrhoea (AOR = 0.89: 95% CI, 0.50–1.58) (*p* = 0.681) and fever (AOR = 1.19: 95% CI 0.78–1.82) (*p* = 0.777) (Table 3).

**Table 3:** Association between maternal vitamin A supplementation and occurrence of common childhood ailments in HIV-positive women, sub-Saharan Africa

Supplementation status	Diarrhoea				Odds ratio (95% CI)	
	Yes		No		Crude	Adjusted^a^
	Freq	%	Freq	%		
Supplemented (*n* = 287)	22	7.7	261	92.3	0.86 (0.50–1.48)	0.89 (0.50–1.58)
Non-supplemented (*n* = 454)	40	8.8	414	91.2	1	1
	**Fever**					
	**Yes**		**No**			
	Freq	%	Freq	%		
Supplemented (*n* = 287)	56	19.5	231	80.5	1.23 (0.84–1.80)	1.19 (0.78–1.82)
Non-supplemented (*n* = 454)	75	16.5	379	83.5	1	1
	**ARI-related symptoms**					
	**Yes**		**No**			
	Freq	%	Freq	%		
Supplemented (*n* = 287)	26	9.1	261	90.9	0.71 (0.43–1.16)	0.65 (0.39–1.10)
Non-supplemented (*n* = 454)	56	12.3	398	87.7	1	1

^a^Adjusted for geographic region of the country, place of delivery, utilization of postnatal care, sex of the newborn, type of household sanitation facility, number of under five children in the household, frequency of watching television, age of the child and vaccination status of the child

## Discussion

This study based on secondary data of multiple DHS conducted in SSA countries, found no statistically significant association between vitamin A supplement provided to HIV-positive women in the postpartum period and, mortality and morbidity from fever, diarrhoea ARI- related symptoms among their breastfed infants.

Vitamin A plays a critical role in the proliferation, regulation and reaction to stimuli of immunocompetent cells [22]. Based on the established knowledge that postpartum VAS improves breastmilk vitamin A concentration [9, 23, 24] and vitamin A status is an important predictor of childhood survival [5], one may deduct that supplement provided to women in the postpartum period boosts the survival of their breastfed infants. Intuitively, the intervention may even seem to be more beneficial to infants born to HIV-positive women because such cases are more liable to VAD [16–18]. However, this study did not come across with such findings. Likewise, a systematic review of three trials conducted in Tanzania, Malawi and Zimbabwe concluded that VAS provided to HIV-positive women during pregnancy or in the postpartum period had no benefit of reducing IMR [25]. Similarly, a systematic review of 14 trials that were not limited to HIV-positive women found no association between postpartum maternal VAS and survival their infants [9].

The unexpected lack of association between postpartum VAS and infant mortality can be explained by a couple of reasons. First, though there is convincing evidence that VAS improves the vitamin A concertation in breast milk, the change in concentration is likely to be modest [9] or may not be sustained beyond the first three or 4 months of supplementation [9, 26–28]. Accordingly, it probably makes little or no contribution to infants’ survival. Further, even though the infants included in study were all breastfeeding during the surveys, or were breastfed until death, the DHS data provides no information about the intensity/frequency of breastfeeding and it is difficult to ascertain whether the infants had been receiving adequate vitamin A via breast milk or not.

The study suggested that VAS given to HIV-positive women in the immediate postpartum period has no association with infants’ morbidity secondary to diarrhoea, fever or ARI-related symptoms. Very few studies have so far investigated the effect of postpartum VAS of HIV-positive women on the pattern of morbidity of their offspring. A randomized controlled trial conducted in Tanzania based on a large sample size (*n* = 1078) concluded that maternal receipt of vitamin A significantly reduced the risk of pneumonia, but had no effect on incidence of diarrhoea [29]. However, a systematic review of multiple trials conducted among apparently health women found no significant contribution of postpartum supplementation for reducing infants’ morbidity [9].

The typical strength of this analysis is that, it is conducted based on the data of reasonably large number of HIV-positive women drawn from multiple SSA countries where VAD has moderate or severe public health significance. Further, considering the fact that the concentration of breast milk retinol becomes less responsive to VAS three or 4 months postpartum [26–28] and the amount of milk infants suck gradually declines after 6 months of age, the study was limited to breastfed infants younger than 6 months of age. We also attempted to control for multiple possible confounders via multivariable regression models.

Nevertheless, the study suffers from multiple methodological limitations. First, in terms of design, the ideal approach to address the research question is through randomized control trials. However, this study employed an observational cross-sectional design that is liable to systematic errors including information bias, selection bias and confounding from extraneous variables. Though we have attempted to adjust for multiple possible confounders via statistical approach, confounding from unmeasured variables or residual confounding due to imprecisely categorized or measured variables, cannot be entirely excluded.

As we used secondary data, it was not possible to account for some crucial variables that had not measured in in the original surveys including HIV status of the infants and progress/stage of the HIV infection in the women. Theoretically, HIV-positive infants and women with advanced HIV infection many benefit more from postpartum VAS than health individuals do. In addition, important information regarding the dosage and exact timing of supplementation was not available; consequently, the analysis was made based on the assumption that the women had received the usual single mega dose (200,000 international unit) supplementation in the first few days after delivery.

Though the study was conducted in SSA where VAD has huge public health significance, it does not mean that all the mother-baby dyads included in the analysis were actually deficient. Therefore, the analysis is liable to ecological fallacy and this could have underestimated the strength of association between the exposure and outcome. It is important to note that the findings cannot be directly generalized to vitamin A deficient HIV-positive women/infants because, at least theoretically, vitamin A deficient subjects are more likely to benefit from the supplement that those with unknown or normal vitamin A status do.

In this study the occurrence of fever, diarrhoea and ARI-related symptoms was only assessed based on self-report of mothers without any supplementary clinical or laboratory investigation. Accordingly, this could have possibly caused misclassification bias and might have resulted in underestimation of the strength of association between the exposure and outcome. A study conducted in rural Bangladesh found that caregivers report has low sensitivity and specificity for diagnosing neonatal illness [30].

## Conclusion

This secondary data analysis observed no statistically significant association between vitamin A supplementation provided to HIV-positive women in the postpartum period and occurrence early infant mortality and morbidity secondary diarrhoea, fever and ARI-related symptoms among their breastfed infants.

## Supplementary information

**Additional file 1.**

**Additional file 2.**

## Data Availability

All data generated or analysed during this study are included in this article.

## Abbreviations

AOR: Adjusted Odds Ratio
ARI: Acute Reparatory Infection
CI: Confidence Intervals
COR: Crude Odds Ratio
DHS: Demographic and Health Surveys
EA: Enumeration Area
HIV: Human Immunodeficiency Virus
IRB: Institutional Review Board
IMR: Infant Mortality Rate
IU: International Unit
SPSS: Statistical Package for Social Science
SSA: Sub-Saharan Africa
VAS: Vitamin A Supplementation
VAD: Vitamin A Deficiency

## References

[1] World Health Organization (2009). Global prevalence of vitamin a deficiency in populations at risk 1995–2005 WHO global database on vitamin a deficiency.

[2] McCauley ME, van den Broek N, Dou L, Othman M (2015). Vitamin a supplementation during pregnancy for maternal and newborn outcomes. Cochrane Database Syst Rev.

[3] Bruins M, Kraemer K (2013). Public health programmes for vitamin a deficiency control. Community Eye Health.

[4] Imdad A, Mayo-Wilson E, Herzer K, Bhutta ZA (2017). Vitamin a supplementation for preventing morbidity and mortality in children from six months to five years of age. Cochrane Database Syst Rev.

[5] Imdad A, Yakoob MY, Sudfeld C, Haider BA, Black RE, Bhutta ZA. Impact of vitamin a supplementation on infant and childhood mortality. BMC Public Health. 2011;S20. 10.1186/1471-2458-11-S3-S20. 10.1186/1471-2458-11-S3-S20 PMC3231894 21501438

[6] West KP, Wu LS, Ali H, Klemm RDW, Edmond KM, Hurt L (2019). Early neonatal vitamin a supplementation and infant mortality: an individual participant data meta-analysis of randomised controlled trials. Arch Dis Child.

[7] World Health Organization and Food and Agriculture Organization of the United Nations (2004). Vitamin and mineral requirements in human nutrition.

[8] Stoltzfus RJ, Underwood BA (1995). Breast-milk vitamin a as an indicator of the vitamin a status of women and infants. Bull World Health Organ.

[9] Oliveira JM, Allert R, East CE (2016). Vitamin a supplementation for postpartum women. Cochrane Database Syst Rev.

[10] Gogia S, Sachdev HS (2010). Maternal postpartum vitamin a supplementation for the prevention of mortality and morbidity in infancy: a systematic review of randomized controlled trials. Int J Epidemiol.

[11] UNAIDS (2019). Global HIV & AIDS statistics — 2019 fact sheet.

[12] UNAIDS. Global HIV & AIDS statistics — 2012 fact sheet. Accessed from: http://files.unaids.org/en/media/unaids/contentassets/documents/epidemiology/2012/gr2012/20121120_FactSheet_Global_en.pdf. Accessed on: Aug 22, 2019.

[13] de Pee S, Semba RD (2010). Role of nutrition in HIV infection: review of evidence for more effective programming in resource-limited settings. Food Nutr Bull.

[14] Singhal N, Austin J (2002). A clinical review of micronutrients in HIV infection. J Int Assoc Phys AIDS Care.

[15] World Health Organization (2010). Antiretroviral therapy for HIV infection in infants and children: towards universal access: recommendations for a public health approach, 2010 revision.

[16] Friis H, Gomo E, Koestel P, Ndhlovu P, Nyazema N, Krarup H (2001). HIV and other predictors of serum beta-carotene and retinol in pregnancy: a cross-sectional study in Zimbabwe. Am J Clin Nutr.

[17] Mulu A, Kassu A, Huruy K, Tegene B, Yitayaw G, Nakamori M (2011). Vitamin a deficiency during pregnancy of HIV infected and non-infected women in tropical settings of Northwest Ethiopia. BMC Public Health.

[18] Moodley D, Moodley J, Coutsoudis A, Coovadia HM, Gouws E (1998). Vitamin a levels in normal and HIV-infected pregnant women. S Afr Med J.

[19] Semba RD, Miotti PG, Chiphangwi JD, Liomba G, Yang LP, Saah AJ (1995). Infant mortality and maternal vitamin a deficiency during human immunodeficiency virus infection. Clin Infec Dis.

[20] Semba RD, Miotti PG, Chiphangwi JD, Dallabetta G, Yang LP, Saah AJ (1998). Maternal vitamin a deficiency and infant mortality in Malawi. J Trop Pediatr.

[21] ICF International (2012). Demographic and health survey: sampling and household listing manual.

[22] Huang Z, Liu Y, Qi G, Brand D, Zheng SG (2018). Role of vitamin a in the immune system. J Clin Med.

[23] Webb AL, Aboud S, Furtado J, Murrin C, Campos H, Fawzi WW (2009). Effect of vitamin supplementation on breast milk concentrations of retinol, carotenoids, and tocopherols in HIV-infected Tanzanian women. Eur J Clin Nutr.

[24] Grilo EC, Lima M, Cunha L, Gurgel C, Clemente HA, Diemenstein R (2015). Effect of maternal vitamin A supplementation on retinol concentration in colostrumEfeito da suplementação materna com vitamina A sobre a concentração de retinol no colostro. J Pediatr.

[25] Wiysonge CS, Ndze VN, Kongnyuy EJ, Shey MS (2017). Vitamin a supplements for reducing mother-to-child HIV transmission. Cochrane Database Syst Rev.

[26] The WHO/CHD Immunization-Linked Vitamin A Group (2002). Vitamin a supplementation of women postpartum and of their infants at immunization alters breast milk retinol and infant vitamin a status. J Nutr.

[27] Bhaskaram P, Balakrishna N, Nair KM, Sivakumar B (2000). Vitamin a deficiency in infants: effects of postnatal maternal vitamin a supplementation on the growth and vitamin a status. Nutr Res.

[28] Rice AL, Stoltzfus RJ, de Francisco A, Chakraborty J, Kjolhede CL, Wahed MA (1999). Maternal vitamin a or beta-carotene supplementation in lactating Bangladeshi women benefits mothers and infants but does not prevent subclinical deficiency. J Nutr.

[29] Fawzi WW, Msamanga GI, Wei R, Spiegelman D, Antelman G, Villamor E (2003). Effect of providing vitamin supplements to human immunodeficiency virus-infected, lactating mothers on the child's morbidity and CD4+ cell counts. Clin Infect Dis.

[30] Choi Y, El Arifeen S, Mannan I, Rahman SM, Bari S (2010). Can mothers recognize neonatal illness correctly? Comparison of maternal report and assessment by community health workers in rural Bangladesh. Tropical Med Int Health.

