# Supplementary material for: Postpartum vitamin A supplementation for HIV-positive women is not associated with mortality and morbidity of their breastfed infants: evidence from multiple national surveys in sub-Saharan Africa
Source: BMC Pediatr. 2020 May 13;20:214. doi: 10.1186/s12887-020-02131-8 (PMC7218630; doi:10.1186/s12887-020-02131-8)
Supplement: Supplementary file 1 — Additional file 1. [file 12887_2020_2131_MOESM1_ESM.docx]

| **Country** | **Year of survey** |
| --- | --- |
| Burkina Faso | 2003 |
| Burkina Faso | 2011 |
| Burundi | 2010 |
| Cameroon | 2004 |
| Cameroon | 2011 |
| Chad | 2014-15 |
| (Republic of) Congo | 2007 |
| (Republic of) Congo | 2013-14 |
| Côte d'Ivoire | 2011-12 |
| Eswatini | 2006-07 |
| Ethiopia | 2005 |
| Ethiopia | 2011 |
| Gabon | 2011 |
| Gambia | 2013 |
| Ghana | 2003 |
| Ghana | 2014 |
| Guinea | 2005 |
| Guinea | 2012 |
| Kenya | 2003 |
| Kenya | 2008-09 |
| Lesotho | 2004 |
| Lesotho | 2009 |
| Lesotho | 2014 |
| Liberia | 2007 |
| Liberia | 2013 |
| Malawi | 2004 |
| Malawi | 2010 |
| Mali | 2006 |
| Mali | 2012-13 |
| Namibia | 2013 |
| Niger | 2006 |
| Niger | 2012 |
| Rwanda | 2005 |
| Rwanda | 2010 |
| Rwanda | 2014-15 |
| São Tomé and Príncipe | 2008 |
| Senegal | 2010-11 |
| Sierra Leone | 2013 |
| Sierra Leone | 2008 |
| Togo | 2013-14 |
| Zambia | 2013-14 |
| Zimbabwe | 2005-06 |
| Zimbabwe | 2010-11 |

Supplementary file 1: List of surveys that met the preliminary inclusion criteria.
